# Anticancer effects of the microbiota: how the microbiome shapes the development of IL-9-producing T cells

**DOI:** 10.1038/s41416-020-0936-1

**Published:** 2020-07-06

**Authors:** Lionel Apetoh

**Affiliations:** 1 INSERM, U1231, Dijon, France; 2 Faculté de Médecine, Université de Bourgogne Franche Comté, Dijon, France

**Keywords:** T cells, Microbiology

## Abstract

IL-9-producing T cells can harbour potent anti-cancer functions. In this issue of the *British Journal of Cancer*, Almeida et al. found that the host microbiota enhances in vivo T cell-derived secretion of IL-9, thereby limiting cancer outgrowth.

## Main

While the ability of transforming growth factor (TGF)-β and interleukin (IL)-4 to induce IL-9 secretion from T cells dates back to 1994,^1^ it was only in 2008 that IL-9-secreting CD4 T cells (T_H_9 cells) were characterised.^2^ Beyond their high secretion of IL-9, T_H_9 cells secrete IL-10 and IL-21 and are highly pro-inflammatory in vivo. Subsequent studies have shown that T_H_9 cells contribute to antitumour immunity, notably in the context of melanoma. Like T_H_9 cells, IL-9-producing CD8 T cells, termed Tc9 cells, were identified as potent effector T cells in vivo. The adoptive transfer of IL-9-producing T cells (T_H_9 or Tc9) cells into tumour-bearing mice leads to cancer elimination in vivo through direct anticancer effects as well as induction of anticancer immune responses.^3–5^ The relevance of these findings has been extended to humans, as underscored by the better clinical prognosis of cancer patients with high IL-9-producing cell infiltration.^6^ The environmental factors responsible for the induction of IL-9-producing T cells in vivo remain elusive.

**Fig. 1:**
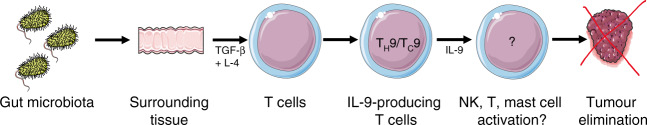
The microbiota harnesses the anticancer functions of IL-9-producing T cells. In tumour-bearing mice, the microbiota drives the induction of IL-9-producing T cells, possibly through favouring TGF-β and IL-4 expression from surrounding tissues. IL-9-producing T cells then exert anticancer effects either by directly eliminating tumour cells or by mobilising additional immune effectors.

In this issue of *British Journal of Cancer*, Almeida et al. uncovered a major contribution of the microbiota in inducing IL-9-producing T cells in mice.^7^ By interrogating the frequency of IL-9-producing T cells in the colonic lamina propria of germ-free or control mice, they found that the former featured a reduced frequency of both T_H_9 and Tc9 cells. Elimination of the microbiota in control mice with antibiotics also led to a reduction in their frequency of colonic T_H_9 and Tc9 cells.^7^ TGF-β and IL-4 were shown to contribute to T_H_9 and Tc9 cell differentiation.^2,3^ Interestingly, Almeida et al. found that antibiotics reduced the expression of TGF-β and IL-4 in the colonic tissue, suggesting that the microbiota supports the generation of IL-9-producing T cells through the induction of these two cytokines (Fig. 1).^7^ Whether the microbiota affects the functions of IL-9-producing T cells was then investigated in the setting of cancer. In the B16F10 mouse melanoma model, the authors found that mice treated with antibiotics featured enhanced tumour progression, which was associated with a lower frequency of IL-9-producing T cells at the tumour site. This could be reversed by the addition of recombinant IL-9 protein, suggesting that IL-9 is the main effector cytokine driving the anticancer effects in the authors’ model (Fig. 1).^7^ IL-9 can be secreted by other cell types such as mast cells, invariant natural killer (NK) T cells and type 2 innate lymphoid cells.^8^ However, similar levels of non-T cells secreting IL-9 were found in tumour-bearing mice irrespective of antibiotic treatment, suggesting that the effects of microbiota are due to T cell-derived IL-9.^7^

Proinflammatory factors have been reported to profoundly affect T differentiation. IL-1β enhances both the differentiation and effector functions of T_H_17 and T_H_9 cells. Likewise, the observations of Almeida et al. that the microbiota supports the generation of IL-9-producing T cells are reminiscent of the known ability of bacteria to affect T_H_17 cell differentiation.^9^ Whether a similar microbial environment promotes IL-17- and IL-9-producing T cell generation was not addressed by Almeida et al. Likewise, how the microbiota supports the anticancer functions of IL-9-producing T cells remains unclear. While IL-9 has been proposed to contribute to CD8 T cell recruitment, the authors found that recombinant IL-9 is not affecting the frequency of CD8 T cell infiltration in the lungs.^7^ However, whether IL-9 administration affects the activation status of innate immune cells such as mast and NK cells, which were shown to contribute to B16F10 tumour elimination, was not investigated (Fig. 1).

While IL-9 was originally shown to promote T cell growth, subsequent studies have demonstrated the pleiotropic functions of IL-9.^8^ One of the notable features of IL-9 is the ability to affect tissue barrier function. In this regard, Renga et al. uncovered that inflammatory dysbiosis can occur in the absence of IL-9,^10^ raising the hypothesis that IL-9 affects the microbiota. Is there a cross-regulation between the microbiota and IL-9 in cancer? While the answer to this question remains unclear at this stage, the study of Almeida et al.^7^ provides evidence that this is a possibility worth investigating.

Although the results of Almeida et al.^7^ in the B16F10 mouse melanoma tumour model still need to be extended to other tumour models and to a human setting, they are raising exciting hypotheses that reach beyond the modulations of the anticancer functions of IL-9-producing T cells. The microbiota indeed not only affects the induction of anticancer immune responses, but was also recently shown to determine the response to anti-programmed cell death protein 1 (anti-PD-1) therapy in multiple cancer types.^11,12^ Likewise, the frequency of circulating T_H_9 cells has been proposed to be associated with the response to anti-PD-1 therapy in humans.^13^ These observations, along with the results of Almeida et al.,^7^ prompt for future studies investigating whether the induction of T cell-derived IL-9 promoted by the microbiota contributes to the anticancer response observed following anti-PD-1 treatment. In summary, Almeida et al.^7^ showed that the development and anticancer functions of IL-9-producing cells can be affected by the microbiota. These results contribute to a better understanding of T cell biology and set bases for novel studies that could possibly unravel novel biomarkers of cancer immunotherapy treatments.

## Data Availability

Not applicable.
